# Effects of Steaming on Fresh Edible Kernels of Waxy and Normal Maize Determined by Metabolomic Analysis

**DOI:** 10.3390/foods13244157

**Published:** 2024-12-22

**Authors:** Yonghui He, Yingjie Zhu, Guangxuan Jiang, Mingyue Xu, Huanhuan Liu, Xuecai Zhang, Zhitong Yin

**Affiliations:** 1 Jiangsu Key Laboratory of Crop Genomics and Molecular Breeding/Key Laboratory of Plant Functional Genomics of the Ministry of Education/Jiangsu Key Laboratory of Crop Genetics and Physiology/Joint International Research Laboratory of Agriculture and Agri-Product Safety of the Ministry of Education, Agricultural College of Yangzhou University, Yangzhou 225009, China; heyonghui@yzu.edu.cn (Y.H.); mx120230806@stu.yzu.edu.cn (Y.Z.); jiangguangxuan123@163.com (G.J.); xumingyuechn@163.com (M.X.); liuhh@yzu.edu.cn (H.L.); 2 Jiangsu Co-Innovation Center for Modern Production Technology of Grain Crops, Yangzhou University, Yangzhou 225009, China; 3 International Maize and Wheat Improvement Center (CIMMYT), Mexico D.F. 06600, Mexico

**Keywords:** maize, cooking, metabolites, waxy maize, kernel

## Abstract

The understanding of the characteristics and metabolite changes in waxy and normal maize kernels after cooking is rather limited. This study was designed to meticulously analyze the differences in characteristics and metabolites of these kernels before and after steaming. To cut environmental impacts, samples were obtained by pollinating one ear with mixed pollen. Non-targeted metabolomics was used to analyze metabolites comprehensively. The results demonstrated that a total of 4043 annotated metabolites were identified. Principal component analysis (PCA) indicated distinct variances between kernels before and after steaming and between the two maize types. Steaming led to an increase in differential metabolites (DEMs) for both maize varieties, noticeably in waxy maize. In waxy maize, the down-regulated DEMs were associated with lipid metabolism, while the up-regulated ones were related to amino acid, phenylpropanoid, and flavone metabolism. Compared to steamed normal maize kernels, waxy maize had more DEMs in purine and steroid pathways, fewer in fatty acid, α-linolenic acid, and phenylpropanoid ones, with marked differences in secondary metabolites like those in amino acid metabolism. This study offers a vital foundation and direction for future research on metabolic pathways regarding maize quality improvement and flavor regulation.

## 1. Introduction

Maize (*Zea mays* L.), a globally crucial staple crop, is fundamental to human nutrition worldwide [1]. It supplies billions with vital calories and nutrients, being a dietary mainstay in many developing areas like sub-Saharan Africa and parts of Latin America, where maize-based foods avert hunger. Rich in carbs, fiber, vitamins (e.g., B3, B1, folate), and minerals (magnesium, phosphorus), it powers daily life and underpins physiological functions [2]. Its consumption across diverse cultures highlights its significance. Notably, maize preparation, especially steaming, is key for product quality, flavor, and nutrition, directly influencing consumer preference and food use [3]. This has driven recent research on cooking-induced changes, especially those during steaming and related physical and metabolic shifts.

In recent years, the study of cooking-induced changes in food materials has grown remarkably. For maize, previous studies have investigated several key areas. For instance, some have examined texture changes during cooking, showing how heat treatment can modify the firmness and chewiness of maize kernels [4], while others have focused on nutrient content, emphasizing potential losses or modifications of vitamins, minerals, and other essential components during the cooking process [5,6,7]. Additionally, research on the alteration of physical properties like texture and macronutrient content during cooking has also been conducted [8,9].

Metabolites, the small molecules crucial in biological processes within maize kernels, can have a significant impact on the final product’s flavor, aroma, and nutritional value when their composition and concentrations change during cooking [10,11,12,13]. Waxy and normal maize varieties have distinct biochemical compositions, with differences in starch structure and other components that likely lead to diverse metabolic responses to the same cooking treatment [6,12]. However, despite prior research, a notable gap persists in grasping the detailed metabolic shifts during waxy and normal maize steaming. Some studies offer partial glimpses, like on texture or nutrient aspects, but lack a full integration of physical and metabolic changes during steaming. Comparisons between waxy and normal maize are also scant, with few exploring how their distinct features impact pathways and responses. Comprehensively examining the characteristics of kernels before and after steaming, quantifying metabolites, and analyzing differential metabolites are conducive to revealing key metabolic pathways and filling these gaps.

Waxy and normal maize, with their distinct starch structures and compositions, are expected to exhibit different behaviors during steaming. To thoroughly investigate the physical and metabolic changes in waxy and normal maize kernels during the steaming process, we explored the general characteristics of the kernels before and after steaming and identified and quantified the metabolites present in the kernels to study the differences between raw and steamed kernels and between the two types of maize. By analyzing the differential metabolites, we revealed the specific metabolic pathways affected by steaming. The main objective of this study is to present a comprehensive picture of the changes during the steaming of waxy and normal maize.

## 2. Materials and Methods

### 2.1. Plant Materials

The experimental materials consisted of two types of maize hybrids. One was Yangbainuo 527, a waxy maize hybrid, and the other was Nongdan 117, a normal maize hybrid. Both were independently bred by Yangzhou University. These maize hybrids were planted in the experimental field of the Agricultural College of Yangzhou University on 15 April 2024. The row spacing during planting was 0.8 m and the plant spacing was 0.25 m. Each plot had an area of 10 m^2^, and a randomized block arrangement was adopted.

For the base fertilizer, slow-release fertilizers represented by Lvjuneng (with N:P_2_O_5_:K_2_O = 27%:9%:9%) were used. During sowing, the base fertilizer was applied to each mu of land in a one-time manner, and the application amount was controlled within the range of 50–60 kg. The field management was the same as that of ordinary large-scale fields, including regular irrigation based on the growth stage of maize and weather conditions to maintain appropriate soil moisture, timely pest and disease control through regular field inspections and the adoption of appropriate biological or chemical control methods when necessary, and effective weed control by combining manual weeding and the appropriate use of herbicides to ensure that the maize plants had sufficient nutrients, water, and light resources during their growth process.

### 2.2. Sample Processing and Sampling

In order to reduce the impact of the environment and the development of ears of different plants on the kernels, we uniformly mixed the pollen of normal maize and waxy maize and then pollinated the female ears of waxy maize. In this way, both waxy maize kernels and normal maize kernels were present on the same ear. After pollination, the pollination time was noted. Based on the growth status of the ears in the field, 25 days after pollination was set as the sampling time.

For fresh sampling, the normal maize kernels in the middle part of three randomly selected ears were mixed together to form one biological replicate of normal maize kernels, and the waxy maize kernels were used as one biological replicate of waxy maize kernels. After sampling, the samples were immediately placed into liquid nitrogen and then stored in a −80 °C refrigerator. Three biological replicates were used in this study.

A similar method was used for sampling for measuring the quality of the steamed maize kernels. The harvested ears had their husks removed, were steamed in boiling water for 20 min, and after being cooled to an edible temperature (about 10 °C), the steamed normal maize kernels and waxy maize kernels samples of three biological replicates were taken, respectively.

### 2.3. Sample Extraction

The samples were ground into an extremely fine powder with a particle size of less than 10 μm in a pre-cooled mortar in liquid nitrogen. To avoid the destruction of metabolites, the whole process, including sample storage and grinding, was carried out under low-temperature conditions. First, 50 mg of the sample was weighed and 1000 μL of the extraction solution containing an internal standard was added (the volume ratio of methanol/acetonitrile/water = 2:2:1 and the concentration of the internal standard is 20 mg/L). Then, the mixture was vortexed for 30 s. Steel beads were added and the sample was processed with a 45 Hz grinder for 10 min, followed by ultrasonication for 10 min (in an ice-water bath). The sample was left to stand at −20 °C for one hour. After, the sample was centrifuged at 12,000 rpm at 4 °C for 15 min. Then, 500 μL of the supernatant was carefully taken out and placed in an EP tube. The extract was dried in a vacuum concentrator. A total of 160 μL of the extraction solution was added (the volume ratio of acetonitrile/water = 1:1) to the dried metabolites for re-dissolution. The sample was vortexed for 30 s and ultrasonication was performed in an ice-water bath for 10 min. The sample was centrifuged at 12,000 rpm at 4 °C for 15 min. Then, 120 μL of the supernatant was carefully taken out and placed in a 2 mL injection vial. An amount of 10 μL from each sample was taken and mixed to form a QC sample for detection on the instrument.

### 2.4. Metabolite Determination

The determination of maize metabolites adopts liquid chromatography–mass spectrometry (LC-MS). Maize contains a rich variety of metabolites, and LC-MS has the capacity to separate and detect both polar and non-polar metabolites. This technique can be utilized to distinguish the differences in metabolites between normal maize and waxy maize kernels. The LC-MS system used for metabolomics analysis consists of a Waters Acquity I-Class PLUS (Waters Corporation, Milford, CT, USA) ultra-high-performance liquid chromatography coupled with a Waters Xevo G2-XS QTOF (Waters Corporation, Milford, CT, USA) high-resolution mass spectrometer. The chromatographic column used is the Acquity UPLC HSS T3 (Waters Corporation, Milford, USA) column (1.8 μm, 2.1 × 100 mm) purchased from Waters. For the positive ion mode (POS) and negative ion mode (NEG), mobile phase A is 0.1% formic acid aqueous solution; mobile phase B is 0.1% formic acid in acetonitrile. The injection volume is 2 μL.

The Waters Xevo G2-XS QTOF high-resolution mass spectrometer is capable of acquiring primary and secondary mass spectrometry data in the MSe mode under the control of the acquisition software (MassLynx V4.2, Waters). In each data acquisition cycle, dual-channel data acquisition for both low-collision energy and high-collision energy can be performed simultaneously. The low-collision energy is turned off, and the high-collision energy range is 10–40 V. The scanning frequency is one mass spectrum per 0.2 s.

The parameters of the ESI ion source are as follows: (1) capillary voltage: 2500 V (positive ion mode) or −2000 V (negative ion mode); (2) cone voltage: 30 V; (3) ion source temperature: 100 °C; (4) desolvation gas temperature: 500 °C; (5) back-blowing gas flow rate: 50 L/h; (6) desolvation gas flow rate: 800 L/h; and (7) the mass-to-charge ratio (*m/z*) acquisition range is 50–1200. Metabolome determination was performed by Biomarker Technologies Co., Ltd. (Beijing, China).

### 2.5. Metabolite Qualitative and Quantitative Analysis

The raw data collected by MassLynx V4.2 were subjected to data processing operations such as peak extraction and peak alignment using Progenesis QI software v4.0. Identification was carried out based on the online METLIN database, public databases, and the self-built library of Biomarker Technologies within the Progenesis QI software. Meanwhile, theoretical fragment recognition was also performed. KEGG classification, Venn diagrams, and heatmap analysis was performed using BMKCloud (www.biocloud.net, accessed on 16 December 2024). PCA plots, correlation plots, and heatmaps were generated using https://www.bioinformatics.com.cn (last accessed on 10 October 2024), an online platform for data analysis and visualization [14].

### 2.6. Statistical Evaluation of the Data

In this study, robust statistical methods were used for reliable results. For maize kernel physical properties, a one-way ANOVA with Duncan’s multiple range test analyzed weight changes. The ANOVA tested if the means of groups (fresh normal, fresh waxy, steamed normal, steamed waxy) differed. With a 0.05 significance level, “a” denoted no significant differences based on these tests. In metabolomics, PCA reduced data dimensionality. PC1 and PC2 showed variance contributions, helping visualize sample group separation. Pearson correlations among replicates ensured data reproducibility, with high positive correlations (*p* < 0.05) validating our methods. For differential metabolite analysis, *t*-tests or an ANOVA with false discovery rate (FDR) correction identified true differences among samples. This avoided false positives from multiple metabolite comparisons. Metabolites passing thresholds were studied for biological roles. Overall, these stats validated findings, ensuring observed changes were meaningful, being biologically and technically relevant.

## 3. Results

### 3.1. General Characteristics of Waxy and Normal Maize Kernels Before and After Steaming

**Figure 1:**
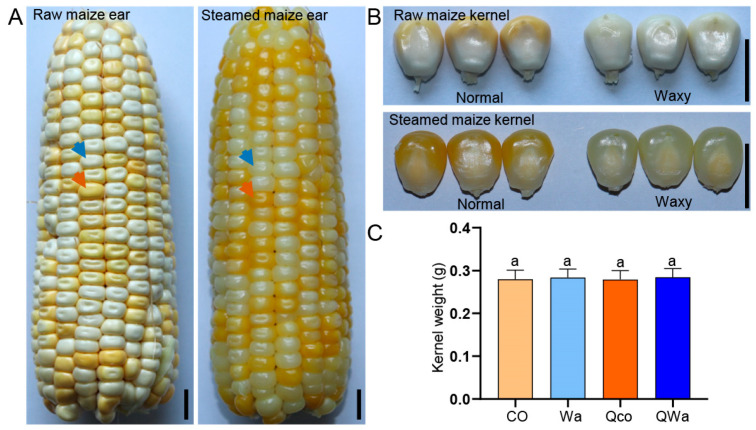
Comparison of characteristics of waxy and normal maize kernels before and after steaming. (**A**) Raw/steamed normal (yellow) and waxy (white) maize kernels from ear 25 days after pollination. Scale bars: 1 cm. Helps observe initial color differences before/after steaming. Blue arrow: waxy corn kernels. Red arrow: normal corn kernels. (**B**) Individual fresh/steamed normal and waxy maize kernels. Scale bars: 1 cm. Picture clearly shows visual transformation from raw to steamed state. (**C**) Weight change in individual waxy and normal maize kernels before and after steaming. “a” indicates no significant differences (>0.05) by Duncan’s test. *n* = 20. CO, fresh normal maize kernels (labeled as control, CO); Wa, fresh waxy maize kernels; Qco, steamed normal maize kernels with respect to kernel quality; Qwa, steamed waxy maize kernels.

In an attempt to minimize the influence of the environment and the development variations in ears among different plants on the kernels, we adopted a specific procedure. We thoroughly and evenly mixed the pollen of normal maize and waxy maize, and then applied this mixed pollen to pollinate the ears of waxy maize. By doing so, both waxy maize kernels and normal maize kernels could be found on the same ear (Figure 1A). Through continuous monitoring of the growth status of the ears in the field, it was identified that the ears 25 days after pollination were at the prime edible stage.

The raw normal maize kernels were yellow in color, while the waxy maize kernels were white. Visually, these two types of kernels shared similar sizes and shapes (Figure 1A). After the steaming process, both the waxy and normal maize kernels became translucent and exhibited a shiny luster. Specifically, the color of the normal maize kernels darkened, and the waxy maize kernels took on a grayish hue (Figure 1B).

To evaluate the potential impact of steaming on the physical properties of the kernels, detailed examinations of the morphology and weight were carried out on fresh normal maize kernels (labeled as control, CO) and waxy maize kernels (Wa), as well as steamed normal maize kernels with respect to kernel quality (Qco) and steamed waxy maize kernels (QWa). The results showed that there were no significant changes in either the shape or the weight of the waxy and normal maize kernels before and after steaming (Figure 1B,C). This indicates that the steaming process within the scope of this experiment had no substantial influence on these particular aspects of the kernels.

### 3.2. Analysis of the Detected Metabolites in Waxy and Normal Maize Kernels

**Figure 2:**
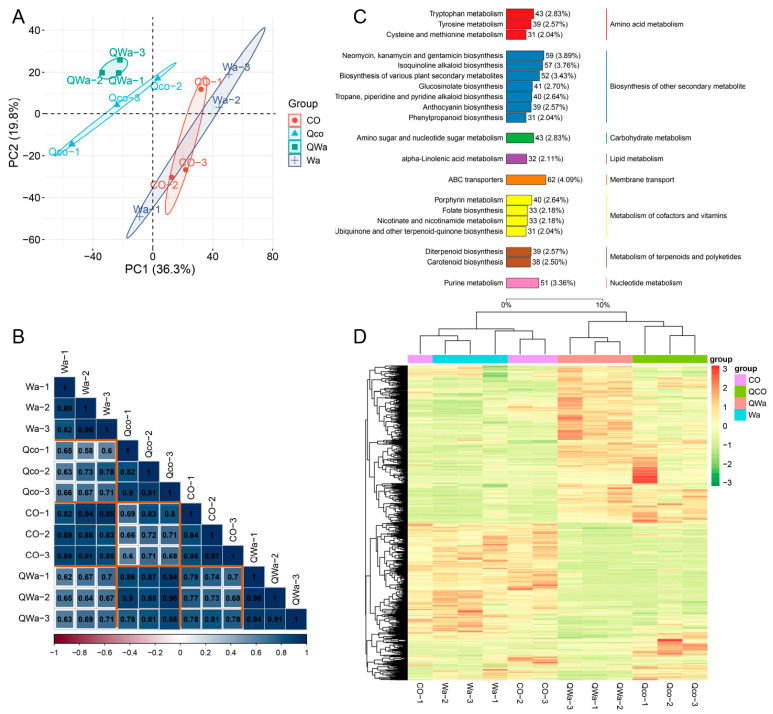
Global analysis of detected metabolites in waxy and normal maize kernels. (**A**) Principal component analysis (PCA) of metabolites in raw normal maize kernels (CO), raw waxy maize kernels (Wa), steamed normal maize kernels (Qco), and steamed waxy maize kernels (QWa). Axes show components and contribution, with points for samples (same group in same color). The dash line indicates the position of zero. (**B**) Correlation plot of metabolites in CO, Wa, Qco, and QWa. Vertical axis for sample names, color for *r* magnitude, replicates with strong positive correlation for data reproducibility. (**C**) Use top 20 KEGG KO pathway level 3 entries to display 4043 metabolites’ classification. Box items denote KEGG pathway annotations, column length shows metabolite numbers in pathways, parentheses figures show proportion. (**D**) Clustering heatmap of 1246 common single differential metabolites (filtered by fold change ≥ 1, VIP ≥ 1, *p*-value ≤ 0.05) in CO, Wa, Qco, and QWa.

To identify the metabolite differences in waxy and normal maize kernels before and after steaming, a non-targeted metabolomics approach was used to conduct metabolic analyses. For the metabolites detected in the default mode, a total of 20,743 peaks were detected, among which 4043 metabolites were annotated (Table S1). The principal component analysis (PCA) score plot showed that PC1 (36.3%) and PC2 (19.8%) separated the four sample groups (Figure 2A). There was little metabolite variation between the fresh waxy and normal maize kernel samples, while there was a clear separation of metabolites in these two types of kernels after steaming. These data suggest that there are metabolic differences between the samples with and without steaming and among different kernel types. A correlation heatmap was constructed, and the Pearson correlation coefficient was used as an evaluation index for the correlation of biological replicates. The results all indicated that there was a highly significant positive correlation among the three biological replicates (Figure 2B).

The integrated metabolic pathway function provided by the KEGG (Kyoto Encyclopedia of Genes and Genomes) database was utilized to annotate all the identified metabolites (Table S2). Subsequently, the top 20 annotations with the greatest number of KO pathway level 3 entries was chosen, and both a summary bar graph and an annotation table (Figure 2C) were created. Among these metabolites, they are predominantly involved in amino acid metabolism, the biosynthesis of other secondary metabolites, carbohydrate metabolism, lipid metabolism, membrane transport, the metabolism of cofactors and vitamins, the metabolism of terpenoids and polyketides, nucleotide metabolism, and so on.

### 3.3. Analysis of Differential Metabolites in Waxy and Normal Maize Kernels Before and After Steaming

**Figure 3:**
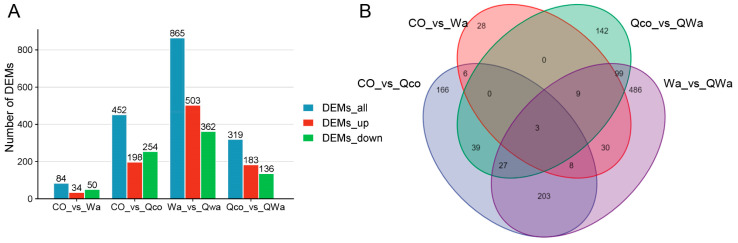
Identification and comparative analysis of differential metabolites in waxy and normal maize kernels pre-/post-steaming. (**A**) Screened all metabolites pairwise by fold change ≥ 1, VIP ≥ 1, *p*-value ≤ 0.05. (**B**) Venn diagram shows common single metabolites in CO_vs_Wa, CO_vs_Qco, Wa_vs_QWa, Qco_vs_QWa groups; overlapping part and numbers mark common metabolites and their quantity. CO, fresh normal maize kernels; Wa, fresh waxy maize kernels; Qco, steamed normal maize kernels; Qwa, steamed waxy maize kernels; DEM, differential metabolites; vs, versus; DEMs_all, all the DEMs in an analytical group; DEMs_up, up-regulated DEMs; DEMs_down, down-regulated DEMs.

For the purpose of identifying the differential metabolites (DEMs) in waxy and normal maize kernels resulting from steaming, all metabolites in pairwise comparisons were screened based on the criteria of fold change ≥ 1, variable importance in projection (VIP) ≥ 1, and *p*-value ≤ 0.05. Altogether, 1246 common single metabolites were identified (Table S3). The clustering heatmap of metabolites also clearly demonstrated the similarities among biological replicates and the differences between the two maize varieties or two treatments (Figure 2D). In the CO_vs_Wa comparison, 84 DEMs were detected (with 34 being up-regulated and 50 down-regulated in Wa). In the CO_vs_Qco comparison, 452 DEMs were found (198 up-regulated and 254 down-regulated in Qco). In the Wa_vs_QWa comparison, 865 DEMs were identified (503 up-regulated and 362 down-regulated in QWa). In the Qco_vs_QWa comparison, 319 DEMs were determined (183 up-regulated and 136 down-regulated in QWa) (Figure 3A). These results demonstrated that there were relatively fewer DEMs between raw waxy maize and raw normal maize. However, a greater number of DEMs were detected after steaming, indicating that the steaming process led to an increase in differential metabolites in the kernels of both maize types. Moreover, compared with the steamed normal maize, the steamed waxy maize contained a larger quantity of up-regulated metabolites. The steaming treatment induced a transformation in the composition of metabolites in maize kernels and generated more DEMs. Notably, the waxy maize kernels exhibited a more significant alteration in metabolites before and after steaming in contrast to the normal maize kernels. This disparity in metabolite alteration might be ascribed to the distinct genetic backgrounds and biochemical compositions of the two maize varieties.

Subsequently, after identifying the differential metabolites in all pairwise comparisons, a further exploration was conducted on the differential metabolites specific to CO_vs_Wa, CO_vs_Qco, Wa_vs_QWa, and Qco_vs_QWa. In the respective comparisons of CO_vs_Wa, CO_vs_Qco, Wa_vs_QWa, and Qco_vs_QWa, there were 28, 166, 486, and 142 unique DEMs (Figure 3B). These results conclusively demonstrated that cooking induced a larger quantity of unique differential metabolites in waxy maize as compared to normal maize, highlighting the differential metabolic responses between the two maize types during the steaming process. The unique DEMs in diverse comparisons, affected by maize types and treatments, correlate with multiple bio-features, determining their quality.

### 3.4. Impact of Steaming on Metabolites in Waxy and Normal Maize Kernels

**Figure 4:**
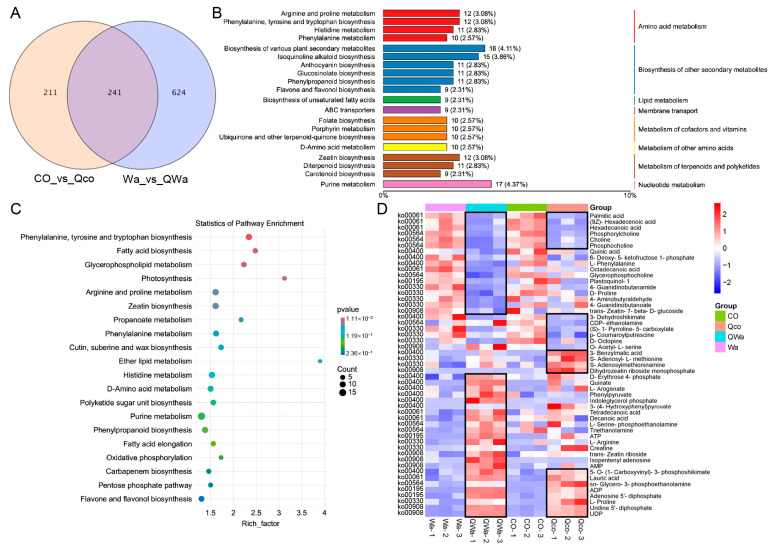
Comprehensive analysis of steaming’s impact on waxy and normal maize kernel metabolites. (**A**) Venn diagram of differential metabolites in CO_vs_Qco and Wa_vs_Qwa. (**B**) Top 20 KO pathway level 3 annotations of 1076 identified differential metabolites using KEGG. (**C**) KEGG—enriched metabolic pathways of metabolites. Analyzing 20 most significantly enriched pathways in DEG metabolites. Each dot represents a KEGG pathway. *X*-axis: enrichment factor (Rich_factor). *Y*-axis: pathway name. (**D**) Heatmap of DEMs in significant metabolic KO pathway in (**C**). Black boxes indicate significant differences compared to the corresponding raw kernels. CO, fresh normal maize kernels; Wa, fresh waxy maize kernels; Qco, steamed normal maize kernels; Qwa, steamed waxy maize kernels.

To analyze the effect of steaming on metabolites within waxy and normal maize kernels, we investigated the common and differential metabolites in normal maize kernels both before and after steaming (CO_vs_Qco), as well as those in waxy maize kernels before and after steaming (Wa_vs_QWa). The Venn diagram demonstrated that there were 1076 differential metabolites in total for CO_vs_Qco and Wa_vs_QWa. Among these, 241 were identical DEMs, accounting for 22.40% (Figure 4A). There were 211 unique metabolites in CO_vs_Qco and 624 in Wa_vs_QWa. Compared to normal maize kernels, waxy maize kernels had a greater number of unique differential metabolites before and after steaming.

KEGG was employed to annotate all 1076 identified differential metabolites (Table S4). The top 20 annotation details with the highest number of KO pathway level 3 entries were selected for generating a summary bar chart and an annotation table (Figure 4B). These metabolites were primarily involved in amino acid metabolism, the biosynthesis of other secondary metabolites, lipid metabolism, and the metabolism of terpenoids and polyketides, among others. KEGG enrichment pathways indicated that the significantly enriched metabolic pathways where the metabolites participated mainly included phenylalanine, tyrosine, and tryptophan biosynthesis (12 DEMs), fatty acid biosynthesis (7), glycerophospholipid metabolism (8), photosynthesis (4), arginine and proline metabolism (12), zeatin biosynthesis (12), etc. (Figure 4C and Table S5). In the significant metabolic KO pathway, the metabolite heatmap revealed that there were six and eight metabolites that were commonly up-regulated and down-regulated, respectively (Figure 4D). The DEMs down-regulated in both waxy and normal maize included palmitic acid, (9Z)-Hexadecenoic acid, and hexadecanoic acid, which belong to the fatty acid biosynthesis (ko00061) pathway, as well as phosphorylcholine, choline, and phosphocholine belonging to the glycerophospholipid metabolism (ko00564) pathway, all of which are associated with lipid metabolism. In contrast, the up-regulated DEMs were distributed among six different metabolic pathways. After steaming, the unique DEMs in waxy maize kernels were mainly concentrated in amino acid metabolism. For example, among the 11 down-regulated metabolites, seven were related to amino acid metabolism, including quinic acid, 6-Deoxy-5-ketofructose 1-phosphate, and L-Phenylalanine belonging to the phenylalanine, tyrosine, and tryptophan biosynthesis (ko00400) pathway, and 4-Guanidinobutanamide, D-Proline, 4-Aminobutyraldehyde, and 4-Guanidinobutanoate belonging to the arginine and proline metabolism (ko00330) pathway. Among the 16 specifically up-regulated DEMs in waxy maize kernels, eight were related to amino acid metabolism, including D-Erythrose 4-phosphate, quinate, L-Arogenate, phenylpyruvate, indoleglycerol phosphate, and 3-(4-Hydroxyphenyl)pyruvate belonging to the phenylalanine, tyrosine, and tryptophan biosynthesis (ko00400) pathway, and L-Arginine and creatine belonging to the arginine and proline metabolism (ko00330) pathway. These data suggest that post-steaming, the commonly down-regulated DEMs in waxy and normal maize link to lipid metabolism, while up-regulated ones spread across more pathways. The unique DEMs in waxy maize kernels focus on amino acid metabolism.

To analyze the KO pathways in which the common and unique metabolites in CO_vs_Qco and Wa_vs_QWa were engaged, we enriched the up-regulated and down-regulated DEMs in each comparison group and categorized them into different pathways (Table 1 and Table 2). The commonly up-regulated DEMs were significantly enriched in zeatin biosynthesis (ko00908), photosynthesis (ko00195), oxidative phosphorylation (ko00190), pyrimidine metabolism (ko00240), purine metabolism (ko00230), ether lipid metabolism (ko00565), etc. (Table 1). Steaming led to an increase in some metabolites within the anthocyanin biosynthesis (ko00942) and isoflavonoid biosynthesis (ko00943) pathways in normal maize kernels, which might be associated with the color of normal maize. In waxy maize kernels, steaming resulted in an increase in some metabolites within amino acid metabolism (including histidine metabolism [ko00340], phenylalanine, tyrosine, and tryptophan biosynthesis [ko00400]), phenylpropanoid biosynthesis (ko00940), and flavone and flavonol biosynthesis (ko00944) pathways. This indicates that steaming raises the contents of some metabolites in the zeatin, pyrimidine, purine, etc., pathways in both waxy and normal maize kernels. Additionally, it increases certain metabolites in the pigment and flavonoid pathways in normal maize and in the amino acid, phenylpropanoid, and flavonoid metabolic pathways in waxy maize kernels.

The commonly down-regulated DEMs were significantly enriched in propanoate metabolism (ko00640), fatty acid biosynthesis (ko00061), glycerophospholipid metabolism (ko00564), cutin, suberine, and wax biosynthesis (ko00073), fatty acid elongation (ko00062), etc. (Table 2). Steaming led to a decrease in some metabolites within the diterpenoid biosynthesis (ko00904) and arginine and proline metabolism (ko00330) pathways in normal maize kernels. In waxy maize kernels, steaming caused a decrease in some metabolites within plant hormone signal transduction (ko04075) and the biosynthesis of unsaturated fatty acids (ko01040) pathways. This shows that steaming reduces the contents of some metabolites in the lipid metabolic pathways in both waxy and normal maize kernels. Moreover, it specifically decreases certain metabolites in the diterpenoid and amino acid metabolic pathways in normal maize kernels and in the hormone and fatty acid synthesis pathways in waxy maize kernels.

### 3.5. Comparison of Differential Metabolites in Waxy and Normal Maize Kernels After Steaming

**Figure 5:**
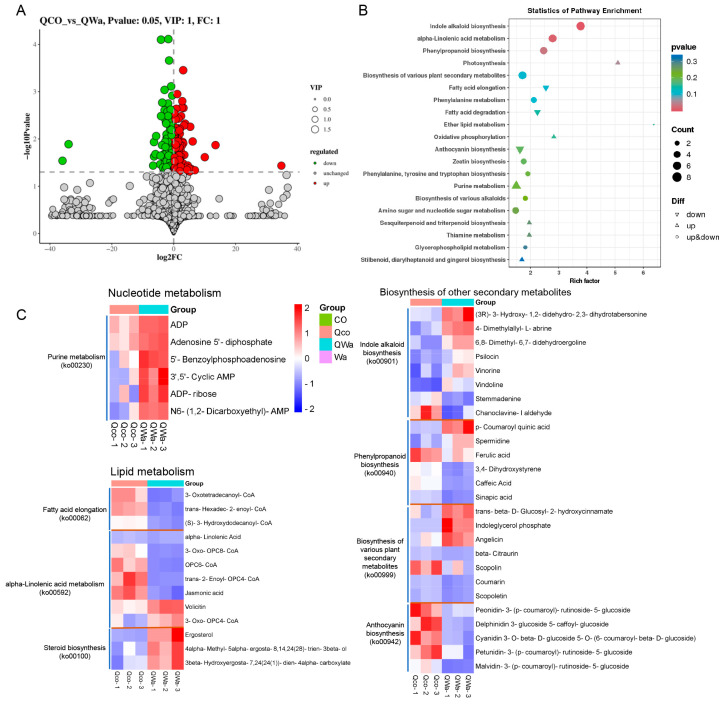
Differential metabolite profiles in steamed waxy and normal maize kernels. (**A**) The comparison of the number of differential metabolites (DEMs) in steamed waxy and normal maize kernels. (**B**) KEGG enrichment pathways of the metabolites between the Qco_vs_QWa. (**C**) Depicts the differences in metabolite abundances in specific metabolic pathways between the waxy and normal kernels after steaming. CO, fresh normal maize kernels; Wa, fresh waxy maize kernels; Qco, steamed normal maize kernels; Qwa, steamed waxy maize kernels; vs, versus.

The steamed waxy maize kernels exhibit a more favorable gustatory perception, which is presumably associated with the differential metabolites present in waxy and normal maize subsequent to the cooking process, as opposed to normal maize. Post-steaming, waxy maize harbors a larger quantity of DEMs (Figure 3A and Figure 5A and Table S6). The KEGG enrichment pathways divulge that the metabolites are predominantly engaged in significantly enriched metabolic pathways such as indole alkaloid biosynthesis, alpha-linolenic acid metabolism, and phenylpropanoid biosynthesis (Figure 5B and Table S7). The up-regulated DEMs are conspicuously enriched in pathways like indole alkaloid biosynthesis (ko00901), photosynthesis (ko00195), and purine metabolism (ko00230), whereas the down-regulated DEMs are principally clustered in pathways including alpha-linolenic acid metabolism (ko00592), anthocyanin biosynthesis (ko00942), fatty acid elongation (ko00062), fatty acid degradation (ko00071), and phenylpropanoid biosynthesis (ko00940, Table 3). It is manifestly discernible that in contrast to the steamed normal maize kernels, the disparities in metabolites of the steamed waxy maize preponderantly center around nucleotide metabolism, lipid metabolism, and secondary metabolism.

The steamed waxy maize possesses a greater abundance of metabolites in metabolic pathways such as purine metabolism (ko00230), steroid biosynthesis (ko00100), and alpha-linolenic acid metabolism (ko00592), as well as a diminished quantity of metabolites in metabolic pathways like fatty acid elongation (ko00062), alpha-linolenic acid metabolism (ko00592), and anthocyanin biosynthesis (ko00942, Figure 5C). In nucleotide metabolism, six metabolites related to purine metabolism, such as ADP, adenosine 5′-diphosphate, 5′-Benzoylphosphoadenosine, 3′,5′-Cyclic AMP, and ADP-ribose, manifest an increment. In lipid metabolism, three metabolites related to fatty acid synthesis, such as 3-Oxotetradecanoyl-CoA and trans-Hexadec-2-enoyl-CoA, display a decrement; three metabolites in the steroid biosynthesis pathway, exemplified by ergosterol, exhibit an augmentation; five metabolites in the alpha-linolenic acid metabolism pathway decrease and two metabolites increase. In secondary metabolites, the metabolites in most metabolic pathways exhibit both upward and downward trends. For instance, five metabolites in the anthocyanin biosynthesis pathway decline, while the preponderant majority of metabolites in the indole alkaloid biosynthesis pathway escalate. Additionally, discrepancies are also observable in metabolites related to amino acid synthesis and other secondary metabolites (Table S6), and these alterations in metabolites might feasibly exert an influence on the flavor of the steamed waxy maize.

## 4. Discussion

### 4.1. Physical and Metabolic Changes in Maize During Steaming

The observed physical and visual changes in waxy and normal maize kernels during steaming align with some existing research on the food processing of cereal kernels. Previous studies have indicated that heat-induced color changes in kernels are often associated with Maillard reactions or other chemical modifications of pigments and related compounds [15]. In our study, the darkening of normal maize kernels and the grayish hue of waxy maize kernels after steaming suggest similar chemical processes at play (Figure 1). The unchanged shape and weight of the kernels within the experimental steaming conditions are consistent with reports suggesting that gentle cooking methods like steaming have minimal impact on the gross physical structure of cereal kernels as opposed to more intense cooking processes such as boiling under high pressure [16]. This stability in physical properties provides a basis for further analysis of the internal chemical changes during steaming.

The significant metabolite variations identified through metabolomics can be better understood in light of our working hypotheses and the previous literature [12,13,17,18]. Our initial hypothesis that steaming would cause distinct metabolic changes in waxy and normal maize was supported by the data (Figure 2, Figure 3, Figure 4 and Figure 5). The separation of metabolite profiles in the PCA score plot between raw and steamed kernels, as well as between the two types of maize, indicates that the steaming process triggers specific biochemical responses (Figure 2). This is in line with previous studies that have shown that cooking can lead to extensive metabolic reprogramming in food materials [10,19]. The involvement of various metabolic pathways, such as amino acid metabolism and the biosynthesis of secondary metabolites, is not surprising considering that these pathways are crucial for the synthesis of flavor- and quality-related compounds in plants [20]. For example, changes in amino acid metabolism can lead to the formation of flavor-enhancing compounds through reactions such as Strecker degradation during cooking [10,21].

### 4.2. Differential Metabolites Unraveling Role in Waxy Maize Cooking and Quality

The identification of differential metabolites provides valuable information regarding the role of specific compounds in the cooking process. The greater number of metabolite changes in waxy maize kernels compared to normal maize kernels might be attributed to the unique biochemical properties of waxy starch. Waxy starch has a different structure and composition compared to normal starch, which could potentially make the waxy maize more susceptible to heat-induced metabolic alterations [12,22,23]. The large number of common single metabolites among different comparisons suggests that there are universal metabolic responses to steaming that are conserved across different maize genotypes (Figure 3B). In CO_vs_Wa, the 84 DEMs (34 up, 50 down in Wa) relate to genetic and physiological differences. Up-regulated ones may aid amylopectin processes in waxy maize, while down-regulated ones are less relevant to its starch metabolism. For CO_vs_Qco’s 452 DEMs (198 up, 254 down in Qco), up-regulated ones could be Qco’s adaptive responses to steaming and down-regulated ones show metabolic changes from normal maize. In Wa_vs_QWa, with 865 DEMs (503 up, 362 down in QWa), up-regulated metabolites are involved in steaming-induced flavor, aroma, and stress response and down-regulated ones are less needed post-steaming. Regarding Qco_vs_QWa’s 319 DEMs (183 up, 136 down in QWa), up-regulated metabolites enhance QWa’s quality and down-regulated ones reflect metabolic shifts. The unique DEMs in different comparisons have distinct biological relevance. In CO_vs_Wa, the 28 unique DEMs may relate to key differences in basal metabolic networks and stress responses between waxy and normal maize. The 166 in CO_vs_Qco are likely tied to specific treatments or genetic variations in Qco, influencing its quality formation and adaptive mechanisms. For Wa_vs_QWa, the 486 unique DEMs are probably associated with the specific quality shaping and metabolic regulation of waxy maize under different treatment conditions during steaming. And the 142 in Qco_vs_QWa reflect the key biological differences between Qco and Qwa in response strategies to environmental factors or processing, which also determine their divergent quality traits in growth and development. These common responses could be targeted in future breeding programs to improve the overall cooking quality of maize. Understanding the functions of these differential metabolites in the context of cooking can help in developing strategies to optimize the flavor and nutritional quality of cooked maize.

The steamed waxy maize kernels present an outstanding taste quality. When compared with normal maize, this enhanced taste can be ascribed to the distinct metabolic alterations that transpire during the cooking procedure. Once steamed, waxy maize kernels manifest a larger quantity of DEMs (Figure 3A and Figure 5A). These DEMs are preponderantly engaged in several vital metabolic pathways, specifically indole alkaloid biosynthesis, alpha-linolenic acid metabolism, and phenylpropanoid biosynthesis (Figure 5B). This distribution within these pathways suggests a potential connection between these metabolic activities and the unique properties of waxy maize kernels [24]. A meticulous examination of the enrichment pathways of these DEMs uncovers fascinating details. The up-regulated DEMs are significantly clustered in pathways such as indole alkaloid biosynthesis, photosynthesis, and purine metabolism. The enrichment within the indole alkaloid biosynthesis pathway, for example, might be responsible for contributing to the unique flavor profile or other favorable characteristics of the waxy maize kernels. Conversely, the down-regulated DEMs are primarily concentrated in pathways including alpha-linolenic acid metabolism, anthocyanin biosynthesis, fatty acid elongation, fatty acid degradation, and phenylpropanoid biosynthesis. The modifications within these pathways during the cooking process are likely to exert an influence on the comprehensive quality of the maize. Alterations in fatty acid-related pathways could potentially impact the texture or mouthfeel experienced during consumption [25]. Similarly, changes in the anthocyanin biosynthesis pathway might have implications for the visual appearance or other associated properties of the maize kernels. These findings suggest that the manipulation of these metabolic pathways through genetic or post-harvest processing methods could lead to improved cooking quality. In addition, the differences in metabolic responses between waxy and normal maize kernels emphasize the need for tailored breeding strategies to optimize the cooking characteristics of different maize types.

### 4.3. Study Limitations and Future Research Directions Laying the Groundwork for Advancement

However, it is important to acknowledge the limitations of this study. First, although we attempted to minimize the influence of environmental and developmental variations by using a specific pollination method, there may still be other external factors that could affect the results, such as soil quality variations within the field or small differences in climate during the growth period. Second, our metabolomics analysis, while comprehensive, may not have captured all possible metabolites due to the limitations of current detection techniques. There could be rare or low-abundance metabolites that play a role in the cooking process but were not identified. Third, the study focused only on the steaming process, and the results may not be directly applicable to other cooking methods, which could cause different physical and metabolic changes in maize kernels. Future research should aim to address these limitations to further enhance our understanding of the complex relationship between maize cooking and its properties. Overall, these findings still provide valuable insights into the physical and metabolic changes during maize cooking, which have implications for understanding flavor improvement and guiding future research in maize processing and breeding.

This study forms a crucial base for future research, chiefly in corn cultivation and food processing. In cultivation, data on waxy and normal maize’s metabolite shifts under steaming equip breeders with targets. Comprehending metabolite–trait links, like those for cooking quality and stress tolerance, enables them to craft targeted strategies. Breeders can then select genotypes with good metabolic changes to breed superior corn. In food processing, analyzing steaming-induced metabolic alterations smoothes the path for optimizing current processes and devising new ones. Processors, using knowledge of pathway modulation, can tweak parameters to control product traits. Also, it may prompt the exploration of post-harvest treatments to mimic or boost beneficial effects, broadening ways to turn corn into quality food ingredients, opening doors for further study and innovation.

## 5. Conclusions

In summary, this study comprehensively analyzed waxy and normal maize kernels before and after steaming. Through specific procedures, we ensured reliable samples. At the prime edible stage, the raw normal maize kernels are yellow and the waxy ones white, both similar in size and shape. Post-steaming, they turn translucent and shiny; the normal kernels darken, and the waxy ones assume a grayish tint. Non-targeted metabolomics analysis detected 20,743 peaks and annotated 4043 metabolites. Metabolomics analysis, including PCA and KEGG pathway analysis, identified significant metabolite differences between fresh and steamed kernels and between the two types. Differential metabolite analysis showed more changes in waxy maize during steaming. Steaming impacted specific metabolic pathways in both types, with unique metabolite changes. The quality of waxy maize after steaming might be associated with the increase in metabolites in metabolic pathways such as purine and steroid metabolism, the decrease in metabolite contents in pathways like lipid metabolism and linolenic acid metabolism, and the changes in secondary metabolites containing amino acids. Focusing on genetically improving maize via breeding strategies that target relevant metabolic pathways, along with devising innovative steaming or preservation techniques to curtail the loss of metabolites, will constitute crucial aspects meriting close attention in future research endeavors. While there may be some environmental and metabolite detection limitations, our results offer crucial insights for improving maize steaming quality and understanding flavor changes in breeding and food processing.

## Figures and Tables

**Table 1:** KEGG pathways associated with the up-regulated DEMs in CO_vs_Qco and Wa_vs_QWa.

Classification	Pathway ID	Pathway Description	MetaboliteRatio	BgRatio	*p* Value
Common DEMs	ko00908	Zeatin biosynthesis	4/36	29/1517	0.004159037
	ko00195	Photosynthesis	2/36	5/1517	0.005236888
	ko00190	Oxidative phosphorylation	2/36	9/1517	0.017756519
	ko00240	Pyrimidine metabolism	3/36	29/1517	0.029369346
	ko00230	Purine metabolism	4/36	51/1517	0.030162404
	ko00565	Ether lipid metabolism	1/36	2/1517	0.046914216
Unique DEMs in CO_vs_Qco	ko00942	Anthocyanin biosynthesis	4/39	39/1517	0.016060433
	ko00943	Isoflavonoid biosynthesis	3/39	24/1517	0.021890918
Unique DEMs in Wa_vs_QWa	ko00340	Histidine metabolism	8/150	28/1517	0.004219737
	ko00400	Phenylalanine, tyrosine, and tryptophan biosynthesis	6/150	20/1517	0.010144848
	ko00940	Phenylpropanoid biosynthesis	7/150	31/1517	0.02753299
	ko00944	Flavone and flavonol biosynthesis	6/150	27/1517	0.043394159

MetaboliteRatio: proportion of target metabolites annotated to a pathway among total target metabolites. Left of diagonal: quantity of KEGG compound IDs of metabolic set annotated to this pathway. Right of diagonal: numbers of KEGG compound IDs of metabolic set annotated to all pathways; BgRatio: proportion of metabolites annotated to background pathway among total background metabolites. Left of diagonal: numbers of annotated background metabolic sets. Right of diagonal: numbers of KEGG compound IDs of background metabolic sets annotated to all pathways; CO, fresh normal maize kernels; Wa, fresh waxy maize kernels; Qco, steamed normal maize kernels; Qwa, steamed waxy maize kernels; DEM, differential metabolites; vs, versus.

**Table 2:** KEGG pathways associated with the down-regulated DEMs in CO_vs_Qco and Wa_vs_QWa.

Classification	Pathway ID	Pathway Description	MetaboliteRatio	BgRatio	*p* Value
Common DEMs	ko00640	Propanoate metabolism	3/33	9/1517	0.000721505
	ko00061	Fatty acid biosynthesis	3/33	11/1517	0.001375565
	ko00564	Glycerophospholipid metabolism	3/33	14/1517	0.002901881
	ko00073	Cutin, suberine, and wax biosynthesis	3/33	18/1517	0.006129505
	ko00062	Fatty acid elongation	2/33	15/1517	0.040384542
Unique DEMs in CO_vs_Qco	ko00904	Diterpenoid biosynthesis	4/42	39/1517	0.020678251
	ko00330	Arginine and proline metabolism	3/42	29/1517	0.043738598
Unique DEMs in Wa_vs_QWa	ko04075	Plant hormone signal transduction	3/90	9/1517	0.013069077
	ko01040	Biosynthesis of unsaturated fatty acids	5/90	28/1517	0.021802619

CO, fresh normal maize kernels; Wa, fresh waxy maize kernels; Qco, steamed normal maize kernels; Qwa, steamed waxy maize kernels; DEM, differential metabolites; vs, versus.

**Table 3:** KEGG pathways associated with the DEMs in QCO_vs_Qwa.

Classification	Pathway ID	Pathway Description	MetaboliteRatio	BgRatio	*p* Value
Up-regulated DEMs	ko00901	Indole alkaloid biosynthesis	6/63	27/1517	0.000602421
	ko00195	Photosynthesis	2/63	5/1517	0.01565681
	ko00230	Purine metabolism	6/63	51/1517	0.016751762
Down-regulated DEMs	ko00592	Alpha-linolenic acid metabolism	5/56	32/1517	0.005373541
	ko00942	Anthocyanin biosynthesis	5/56	39/1517	0.012611934
	ko00062	Fatty acid elongation	3/56	15/1517	0.015820647
	ko00071	Fatty acid degradation	3/56	17/1517	0.022439355
	ko00940	Phenylpropanoid biosynthesis	4/56	31/1517	0.02494534

Qco, steamed normal maize kernels; Qwa, steamed waxy maize kernels; DEM, differential metabolites; vs, versus.

## Data Availability

The National Genomics Data Center (https://ngdc.cncb.ac.cn/, accessed on 16 December 2024) provides metabolomics data under accession PRJCA033036. The original contributions presented in the study are included in the article and supplementary materials, further inquiries can be directed to the corresponding author.

## Supplementary Materials

The following supporting information can be downloaded at: https://www.mdpi.com/article/10.3390/foods13244157/s1, Table S1: Qualitative and quantitative analysis of 4043 metabolites. Table S2: The annotation categories of the KEGG database for all annotated metabolites. Table S3: Identified differential metabolites in CO_vs_Wa, CO_vs_Qco, Wa_vs_QWa, and Qco_vs_QWa. Table S4: Differential metabolites in CO_vs_Qco and Wa_vs_QWa. Table S5: KEGG pathway enrichment analysis of all identified differential metabolites in CO_vs_Qco and Wa_vs_QWa. Table S6: Differential metabolites in Qco_vs_QWa. Table S7: KEGG pathway enrichment analysis of differential metabolites in Qco_vs_QWa.
